# Vis LED Photo-Fenton Degradation of 124-Trichlorobenzene at a Neutral pH Using Ferrioxalate as Catalyst

**DOI:** 10.3390/ijerph19159733

**Published:** 2022-08-07

**Authors:** Leandro O. Conte, Carmen M. Dominguez, Alicia Checa-Fernandez, Aurora Santos

**Affiliations:** 1 Chemical Engineering and Materials Department, Chemical Sciences Faculty, Complutense University of Madrid, 28040 Madrid, Spain; 2 Instituto de Desarrollo Tecnológico para la Industria Química (INTEC), Consejo Nacional de Investigaciones Científicas y Técnicas (CONICET) and Universidad Nacional del Litoral (UNL), Santa Fe 3100, Argentina

**Keywords:** Photo-Fenton, chlorinated organic compounds, 124-Trichlorobenzene, visible LED, neutral pH, ferrioxalate

## Abstract

Chlorinated organic compounds (COCs) are among the more toxic organic compounds frequently found in soil and groundwater. Among these, toxic and low-degradable chlorobenzenes are commonly found in the environment. In this work, an innovative process using hydrogen peroxide as the oxidant, ferrioxalate as the catalyst and a visible light-emitting diode lamp (Vis LED) were applied to successfully oxidize 124-trichlorobenzene (124-TCB) in a saturated aqueous solution of 124-TCB (28 mg L^−1^) at a neutral pH. The influence of a hydrogen peroxide (HP) concentration (61.5–612 mg L^−1^), Fe^3+^ (Fe) dosage (3–10 mg L^−1^), and irradiation level (Rad) (I = 0.12 W cm^−2^ and I = 0.18 W cm^−2^) on 124-TCB conversion and dechlorination was studied. A D–Optimal experimental design combined with response surface methodology (RSM) was implemented to maximize the quality of the information obtained. The ANOVA test was used to assess the significance of the model and its coefficients. The maximum pollutant conversion at 180 min (98.50%) was obtained with Fe = 7 mg L^−1^, HP = 305 mg L^−1^, and I = 0.12 W cm^−2^. The effect of two inorganic anions usually presents in real groundwater (bicarbonate and chloride, 600 mg L^−1^ each) was investigated under those optimized operating conditions. A slight reduction in the 124-TCB conversion after 180 min of reaction was noticed in the presence of bicarbonate (8.31%) and chloride (7.85%). Toxicity was studied with Microtox® (Azur Environmental, Carlsbad, CA, USA) bioassay, and a remarkable toxicity decrease was found in the treated samples, with the inhibition proportional to the remaining 124-TCB concentration. That means that nontoxic byproducts are produced in agreement with the high dechlorination degrees noticed.

## 1. Introduction 

The occurrence of chlorinated organic compounds (COCs) in wastewater, surface water, and groundwater environments has become an emerging public and scientific concern due to their potential adverse impacts on biota and human health. Because of their high persistence in the environment and their toxic, carcinogenic, and hydrophobic characteristics, several COCs (chloroethanes, chlorophenols, chlorobenzenes, etc.) are listed as priority substances by the EU Water Framework Directive [1]. Trichlorobenzenes (TCBs) are synthetic chemicals extensively used in synthesizing pesticides, repellents, dyes, solvents, etc. [2,3,4]. Furthermore, TCBs are byproducts of highly chlorinated pesticide degradation, such as hexachlorocyclohexanes [5,6,7]. Consequently, they have become ubiquitous pollutants [8,9], being detected (concentration from ng L^−1^ to mg L^−1^) in water, soil, and sediments [10]. 

Due to their high chemical stability and low biodegradability, TCBs are refractory to degradation using conventional physicochemical or biological processes, and the development of more effective treatments is required. Adsorption was an effective method to remove TCBs [11,12,13] but only transferred the pollution to other media that became waste. In this context, Advanced Oxidation Processes (AOPs) based on the generation of highly reactive species, such as hydroxyl or sulfate radicals (OH^•^ or SO_4_^−•^), are efficient remediation methods [14,15,16]. The oxidation of TCBs with sodium persulfate [17], Fenton’s reagent [18], ozone [19], photocatalysis [20,21], electrochemical processes [22], and catalytic wet oxidation (CWO) [23,24] have been studied. More recently, UV light has been proven to enhance TCB oxidation by persulfate [25] or hydrogen peroxide [15]. 

Among the AOPs, processes using Fenton reagent (hydrogen peroxide and iron salts) have multiple advantages, such as high efficiency, lack of high equipment requirements, and reagent availability. Their main limitation lies in the narrow acidic pH range (2.8–3.5) needed for the optimal application of the process [26,27]. Furthermore, additional post-treatments (i.e., neutralization, separation, and management of the iron hydroxide sludge generated) are often required [28]. CWO and electrochemical processes have the drawback of high energy requirements. UV radiation with photocatalyst or with oxidants promotes radical species generation. UV can split the oxidant (H_2_O_2_ and S_2_O_8_^2−^) to generate hydroxyl (OH^•^) or sulfate (SO_4_^−•^) radicals. Moreover, UV improves the photo-assisted reduction of Fe^3+^ to Fe^2+^ [27,29]. Fe^2+^ is the catalyst or activator using hydrogen peroxide or persulfate, respectively. Disinfection with UV is often quite cost-/energy-efficient compared to other disinfection methods, as only low UV doses are usually required. However, using UV as Advanced Oxidation Processes in the abatement of recalcitrant organic pollutants often has a high energy consumption as the main drawback. 

The combination of the Fenton process with UV-Vis (photo-Fenton reaction) also leads to a high pollutant degradation rate, associated with increased iron catalytic cycle reaction rates [30,31]. However, expensive and high energy-consuming light sources, such as Hg or Xe lamps [32], are often used. Light-emitting diode (LED) lamps emerge as a promising alternative in this scenario. These lamps present many benefits, such as low power consumption, electrical stability, durability, high photon efficiency, and the possibility of emission at selective or more exhaustive wavelengths [33,34]. Despite all this, there are very few works in the bibliography in which the feasibility of the photo-Fenton process using only Vis light is studied [35,36,37,38]. 

Moreover, some limitations of the iron salts commonly used in the “traditional” photo-Fenton process are the narrow pH range at which they should be operated (close to 2.8) and their low molar absorption coefficients in the UV-Vis spectrum region. At higher pH values, the concentration of the catalyst decreases because of its precipitation as iron hydroxides. The use of complexing agents (oxalates, citrates, ethylenediamine-N, N′-succinic acid (EDDS), etc.) allows for operating at pHs close to neutrality [39]. These compounds form complexes with the catalyst at a neutral pH, keeping it soluble and extending the applicability of the photo-Fenton treatment to a broader pH range [40]. Iron (III) complexes (iron-oxalate, iron-citrate, iron-EDDS, etc.) have typically higher molar absorption coefficients in the UV-Vis regions than the iron (III) cation [41,42,43]. Therefore, its use is beneficial in saving energy. 

This work studied the abatement of 124-TCB by the photo-Fenton process at a neutral pH using a ferrioxalate complex, such as an iron source. A commercial Vis LED lamp was used as an innovative, efficient, and cheaper light source. To our knowledge, this is the first time that the reduction of 124-TCB with hydrogen peroxide, oxalate–iron complex, and Vis LED lamp has been studied.

The effect of several variables (oxidant and catalyst concentration, irradiation level, pH, temperature, etc.) on the photo-Fenton efficiency process must be investigated. To evaluate all of them reliably, with a lower number of experiments, Experimental Designs (EDs) are proposed. ED is a statistical tool used to reduce the number of experiments (optimizing time and cost) and maximize the quality of the information obtained [44,45,46], providing valuable information for extrapolating the experimental conditions to pilot plant scale reactors [42,47].

The effect of the three main reaction variables: irradiation level (intensity), hydrogen peroxide concentration, and iron dosage on 124-TCB conversion on the pollutant conversion and dechlorination were evaluated. The effect of two anions (${\mathrm{HCO}}_{3}^{-}{\;\mathrm{and}\;\mathrm{Cl}}^{-},\;600{\;\mathrm{mg}\;L}^{-1}$) commonly present in real water matrices on the pollutant abatement was studied. Moreover, the toxicity evaluation of initial and final reaction samples was measured through the Microtox^®^ test. The results give practical information to design a future real large-scale process implementation. 

## 2. Material and Methods

### 2.1. Chemicals

124-TCB (Sigma-Aldrich, Darmstadt, Germany, ≥99%) was used as the target pollutant. The ferrioxalate solution, the catalyst of the process, was prepared using potassium oxalate monohydrate (Sigma-Aldrich, 99.50%) and iron (III) sulphate hydrate (Sigma-Aldrich, 97%). Hydrogen peroxide (35 wt.%), titanium oxysulfate (used for the quantification of hydrogen peroxide), sodium carbonate, sodium bicarbonate, sulfuric acid, oxalic acid, acetone (used for Ionic Chromatography analysis), 1,10-phenanthroline, sodium acetate (used in the measurement of iron in solution), n-hexane, tetrachloroethane, butyl cyclohexyl (used in COC determination by GC), NaOH (for pH adjustment), catalase, sodium bicarbonate, and sodium chloride were all purchased from Sigma-Aldrich. The bacteria *Vibrio fischeri* (Microtox^®^ Acute Reagent, Azur Environmental, Carlsbad, CA, USA) was supplied by I.O. Analytical. All the stock solutions and their dilutions were prepared with high-purity water from a Millipore Direct-Q system (Millipore Corporation, Burlington, MA, USA) (resistivity >18 MΩ cm at 25 °C).

### 2.2. Experimental Device and Procedure

The experiments were carried out in a double-walled cylindrical thermostat batch reactor (see Figure S1 in the Supplementary Materials, volume reactor V_T_ = 0.10 L). The liquid in the reactor was stirred using a magnetic plate (IKA C-MG HS 7, Staufen, Germany). A jacket was used to maintain isothermal conditions (T = 25 °C). The reaction mixture was illuminated with a high-power collimated LED of Mightex (LCS-0470-50-11, Lasing, Spain) at the top of the reactor. The emission peak of the led lamp was at 470 nm, representative of the visible fraction of the incident terrestrial solar radiation spectrum. In addition, it is close to the wavelength of the sun’s peak (between 483 and 502 nm) radiation intensity [48,49]. The collimator light source produces an optical power output of up to 4.17 W, and the power can be manually adjusted using a Mightex LED controller. The LED source emits over a surface reaction of 11 cm^2^ (reactor window). Lorenzo et al. [50] give more details about the reaction device.

Firstly, a ferrioxalate solution was prepared according to the methodology described by [51]. The required volumes of concentrated acidic (pH = 4) solutions of Fe^3+^ (250 mg L^−1^) and oxalate (2500 mg L^−1^) were mixed with distillate water to reach the Fe^3+^ concentration desired in the experiment, and a molar ratio of Fe^3+^: oxalate 1:10, to ensure that the iron added was completely complexed as $Fe^{III}{\left(C_{2}O_{4}\right)}_{3}^{3-}$(Visual MINTEQ version 3.0, USEPA, EPA, USA). The pH was then adjusted to neutral (pH = 7). This organic complex has higher molar radiation absorption coefficients in the UV-Vis region than the aqueous iron complexes [41]. After that, 124-TCB was added to saturate the ferrioxalate complex in the pollutant (28 mg L^−1^), and the pH was adjusted to pH = 6.5 (using a concentrated NaOH solution). The concentration of 124-TCB was high enough to ensure it is in the range of values found in groundwater polluted with these compound spills [52]. Inorganic anions, when applicable, were also added to this solution of ferrioxalate complex. A volume of 100 mL of this solution was placed in the reactor. After adding the oxidant (hydrogen peroxide, HP), the first sample (zero time) was immediately taken, and the lamp shutter was removed, starting the reaction. Several samples (4 mL) were taken at different times to study the pollutant and oxidant evolution during the reaction time. 

### 2.3. Analytical Methods

The irradiance and radiation flux of the LED lamp were measured using a Flame UV-Vis spectrometer coupled with a monacolin optical fiber of 100 µm in diameter using a cosine corrector (Ocean Insight, Duiven, The Netherlands). The system was calibrated using an HL-3P-CAL lamp (Ocean Insight, Duiven, The Netherlands). More details can be found elsewhere [50]. Text S1 and Table S1 were added in the Supplementary Materials to give more details about the irradiance measured.

Chlorinated Organic Compounds, COCs, were identified by Gas Chromatography (Agilent, Santa Clara, CA, USA) with a Mass Spectrometry Detector ((GC/MS). The (residual) 124-TCB and degradation products/formed COCs were quantified using a GC (Agilent, USA) with a Flame Ionisation Detector (GC/FID) and an Electron Capture Detector (GC/ECD). A chromatographic column HP-5MS ((5%-phenyl)-methyl polysiloxane, 30 m × 0.25 mm ID × 0.25 μm) was used as a stationary phase in both GC, and a constant flow rate of helium was used as a mobile phase (1.7 mL min^−1^). The chromatographic oven worked under a programmed temperature gradient (starting at 80 °C with a temperature ramp of 18 °C min^−1^ up to 180 °C, then kept constant for 15 min). Butyl cyclohexyl and tetrachloroethane were added to the hexane extracts as internal standards (ISTDs) for GC analyses to reduce experimental errors in COC quantification. Further details of the analytical methods used in the present work have been reported elsewhere [50,52]. Previously to GC analysis, the aqueous phase (4 mL) was extracted and concentrated with n-hexane (0.8 mL). The mixture of both phases was shaken, followed by settlement, allowing the organic phase separation by decantation. The concentrations of HP and Fe were determined by colorimetric titration (spectrophotometer BOECO S-20 UV-Vis, Germany) at 410 nm and 510 nm, respectively [41]. The pH was measured with a Basic 20-CRISON (Barcelona, Spain) electrode. Additionally, short-chain carboxylic acids and chlorides were measured by Ion Chromatography (Metrohm, Herisau, Switzerland) with anionic chemical suppression and a conductivity detector, using a SUPP5 5–250 column (25 cm length and 4 mm diameter) and Na_2_CO_3_ (3.2 mM) and NaHCO_3_ (1 mM) as mobile phase (0.7 mL min^−1^). A solution of acetone and sulfuric and oxalic acids was used to regenerate the ionic resins. 

A Microtox^®^ 500 Toxicity Analyser (Azur Environmental, Carlsbad, CA, US) was used to determine the toxicity of the samples during the photo-Fenton process. The toxicity was measured as the light emission inhibition percentage (bioluminescence assay) of the *Vibrio fischeri* bacteria (Microtox Acute Reagent) after 15 min of incubation [53]. Solvent controls were performed to determine the inhibition obtained with the aqueous ferrioxalate solution (higher Fe concentration used 10 mg L^−1^) and the Diluent Solution (NaCl 2%). Measurements of light inhibition were obtained in triplicate, without sample dilution, and differences higher than 15% were discarded, and the measurement was repeated. The pH of the samples before the toxicity measurements was measured to control that it was between 6 and 8, and hydrogen peroxide remaining in the aqueous sample was decomposed to water and oxygen before analysis using catalase (2000 mg L^−^^1^ of >3000 U mg^−^^1^ bovine liver). 

### 2.4. Experimental Design

In this work, the variables studied were hydrogen peroxide concentration (HP), iron concentration (Fe), and level of irradiation (Rad). The iron concentration range was selected considering the maximum iron concentration allowed by Spanish law (Ley 5/2002 Spain) in water effluents before discharge (3–10 mg L^−1^); the initial molar ratio of oxalate/iron was set to 10 to ensure the iron oxalate complex was formed [54]. The hydrogen peroxide concentration was selected from the stoichiometric amount theoretically required to mineralize 124-TCB according to Equation (1). Therefore, the HP concentration range selected was between 61.50 and 615 mg L^−1^, corresponding to 1 and 10 times the theoretical stoichiometric values needed for the complete mineralization of 28 mg L^−1^ of 124-TCB, respectively.

(1) $$C_{6}H_{3}Cl_{3}\left(124-TCB\right)+12\;H_{2}O_{2}\;\overset{\;}{\to }6\;CO_{2}+3\;HCl+12\;H_{2}O\;$$

Irradiation level (Rad) was selected from 0.12 to 0.18 W cm^−2^, based on previous works with the same LED lamp [50]. The real lamp power (P) is close to its nominal (Pn) value over the reactor window. Two irradiation levels were evaluated: Low (Pn = 1.04 W; P = 1.01 W) and High (Pn = 2.07 W; P = 1.95 W). In addition, local radiation fluxes averaged over the reactor window (q_W_) were measured (between 400 and 500 nm) using a Flame UV-Vis spectrometer (Ocean Insight, Duiven, The Netherlands). The following values were obtained: q_W_ = 4.40 × 10^−7^ E cm^−2^ s^−1^ (0.12 W cm^−2^) for Low Rad and q_W_ = 7.89 × 10^−7^ E cm^−2^ s^−1^ (0.18 W cm^−2^) for High Rad conditions. Dark reaction conditions (I = 0 W cm^−2^) were also tested. 

A D-Optimal experimental design was constructed to choose the experimental conditions of the runs, minimizing the variance of the estimated regression coefficients through optimality criteria. This model generates a possible set of runs inside the design space defined and can be used when numeric (Fe and HP) and categorical (Rad) factors are present [42,55]. From the design proposed, the response surface methodology (RSM) [56,57] was used to study and optimize 124-TCB degradation through photo-Fenton processes. The maximum 124-TCB conversion obtained in the dark reactions was less than 10% after 180 min. Therefore, this condition of Rad was not included in the proposed design of experiments. Moreover, it was experimentally found that, at the conditions of run N3, the 124-TCB conversion obtained at 180 min was lower than 7% in the absence of irradiation, catalyst, or oxidant (data not shown).

The experimental design matrix obtained by D-Optimal, including the experimental conditions of the studied parameters and the response obtained (pollutant and oxidant conversions and dechlorination degree), are present in Table 1 (23 experiments, Run N1–N23). The number of tests to be carried out and combinations of levels for each variable studied and the necessary replicas are defined by the computer algorithm. Moreover, the analysis of variance (ANOVA) was used to evaluate the obtained models statistically. 

The influence of these parameters on the 124-TCB and HP conversions and dechlorination degree at 180 min of reaction was evaluated. The dechlorination degree $\left(Cl^{-}/Cl_{0}{}^{180\;\min}\right)$ was calculated as the ratio of chloride in solution (mg L^−1^) at 180 min to the chloride in the initial 124-TCB concentration (16.4 mg L^−1^).

Moreover, three additional experiments were conducted to analyze the effect of common anions in groundwater on pollutant conversion and oxidant consumption. The first was free of chloride and bicarbonate; the second was accomplished by adding chloride (600 mg L^−1^), and the third was conducted by adding bicarbonate (600 mg L^−1^). Reaction conditions were selected for those giving the maximum 124-TCB conversion (0.18 W cm^2^, 290 mg L^−1^HP, and 7.10 mg L^−1^ Fe^3+^). The anion concentrations selected here are experimentally found in groundwater highly contaminated with chlorinated organic compounds [52].

## 3. Results and Discussion

The results for 124-TCB and HP conversions and dechlorination degree at 180 min are also summarized in Table 1. The following sections discuss the influence of the variables studied on the pollutant and oxidant conversions and dechlorination achieved.

It should be noted that the pH was kept within the range 6–7 in all cases during the time interval studied (data not shown), which is an essential operational advantage since it demonstrates the feasibility of the process for this condition, avoiding after-treatment pH adjustments.

### 3.1. Influence of the Irradiation Level

**Figure 1:**
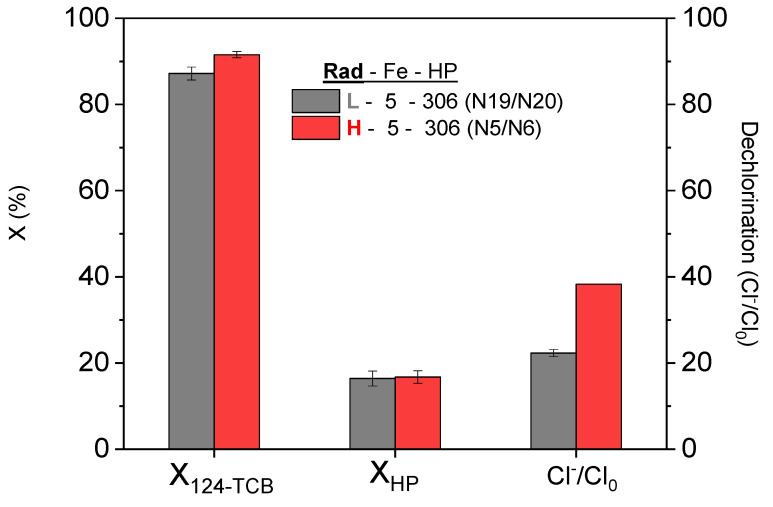
Effect of the irradiation level on the conversion of 124-TCB and HP and dechlorination degree achieved after 180 min of reaction. Experimental conditions: Fe = 5 mg L^−1^, H_2_O_2_ = 306 mg L^−1^, 124-TCB = 28 mg L^−1^, Low rad intensity (L), and High rad intensity (H).

As shown in Table 1, the influence of radiation on the process was significant. Lower 124-TCB conversions were obtained using Low Rad than High Rad for the studied range of operating conditions. However, at the highest hydrogen peroxide concentration and iron concentration used (612 mg L^−1^ and 10 mg L^−1^, respectively), the 124-TCB conversion is lower at the higher irradiation level used (as can be deduced from the comparison of runs N4 and N15. This finding can be due to the unproductive reactions occurring at these high concentrations of the oxidant and catalyst, enhanced by the radiation. However, at the highest hydrogen peroxide concentration and iron concentration used, the 124-TCB conversion is lower at the lower radiation (as can be deduced from a comparison of the runs N4 and N15. This can be due to the unproductive reactions occurring at these high oxidant and catalyst concentrations, enhanced by the high radiation level. Figure 1 shows the pollutant and oxidant conversions and the dechlorination degree achieved after 180 min of reaction at an initial HP concentration of 306 mg L^−1^ and Fe(III) initial concentration of 5 mg L^−1^ for both irradiation levels (High: Run N5/N6 and Low: Run N19/N20). These reaction conditions (Fe and HP concentrations) correspond to the central point of the proposed design of the experiments, and these runs were performed in duplicate. For a similar oxidant conversion (16.75% vs. 16.42%), a higher conversion of the contaminant (91.58 vs. 86.90) and dechlorination degree (37.45% vs. 22.31 %) was reached using High Rad conditions. The difference is especially noticeable for the dechlorination achieved.

It is also worth mentioning that for dark conditions (Fenton) and same reaction conditions (Fe = 5 mg L^−1^, H_2_O_2_ = 306 mg L^−1^, and 124-TCB = 28 mg L^−1^), the maximum conversions of the contaminant reached after 180 min of reaction do not exceed 10%. Therefore, the significance of radiation in the process is very relevant. Furthermore, the radiation source used in this research corresponds to the range of visible wavelengths of the radiation spectrum (470 nm). Then, the process’s applicability using the sun as a radiation source would be very promising (the sun’s peak wavelength is between 483 and 502 nm).

### 3.2. Influence of the Catalyst Concentration

**Figure 2:**
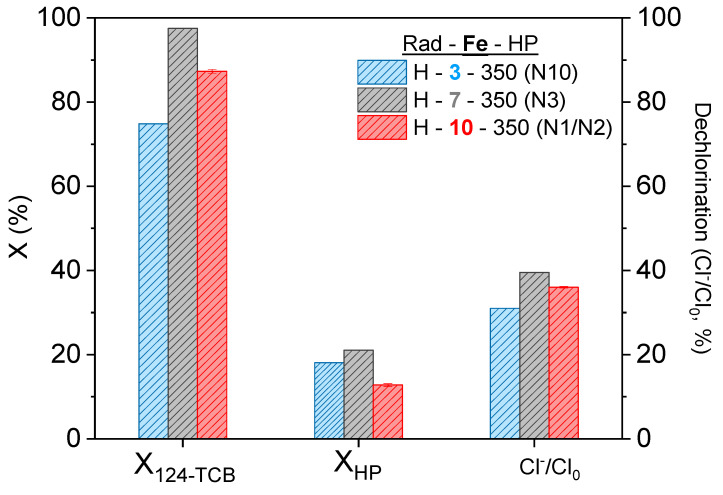
Effect of catalyst concentration on the conversion of 124-TCB and HP and the dechlorination degree achieved after 180 min of reaction. Experimental conditions: H_2_O_2_ = 350 mg L^−1^, 124-TCB = 28 mg L^−1^, and High rad intensity (H).

The effect of different catalyst dosages (Fe = 3–10 mg L^−1^) was analyzed considering a central concentration of oxidizing agent (HP = 350 mg L^−1^) and the highest radiation studied. The results from runs N1, N2, N3, and N10 are shown in Figure 2. Maximum conversions of contaminant and dechlorination degree at 180 min were obtained in run N3 (7 mg L^−1^ of iron concentration) $X_{124-TCB,exp\;}^{180\;\min}=98.50\%$ and $Cl^{-}/Cl_{0}{}^{180\;\min}=39.54\%$. At the lowest catalyst concentration used (Run N10, Fe = 3 mg L^−1^), the pollutant conversion ($X_{124-TCB,exp\;}^{180\;\min}=75.45\%$) was significantly diminished, confirming that a low catalyst dosage limits the radical generation (Equations (2)–(4)). However, an inhibitory effect of the catalyst concentration (lower contaminant conversion and dechlorination degree) was observed when using the highest iron dosage. This inhibitory effect noticed at a high iron concentration can be due to the free radical unproductive consumption of hydroxyl radicals by Fe(II) shown in Equation (5) [58]. Similar conversions (about 20%) of the oxidant were achieved in the three runs in Figure 2 for the three iron dosages.

(2) $$Fe^{\left(III\right)}{\left(C_{2}O_{4}\right)}_{3}{}^{3-}\overset{\;h\nu \;\;\;}{\to }Fe^{II}+2C_{2}O_{4}^{2-}+C_{2}O_{4}{}^{-}$$

(3) $$Fe\left(II\right)+H_{2}O_{2}\overset{\;\;\;\;}{\to }Fe\left(III\right)+OH^{-}+OH\cdot$$

(4) $$Fe\left(III\right)+3C_{2}O_{4}{}^{2-}\overset{\;K_{eq}\;}{⇄}Fe^{\left(III\right)}{\left(C_{2}O_{4}\right)}_{3}{}^{3-}$$

(5) $$Fe\left(II\right)+OH\cdot \;\to Fe^{\left(III\right)}+OH^{-}$$

### 3.3. Influence of the Oxidant Concentration

**Figure 3:**
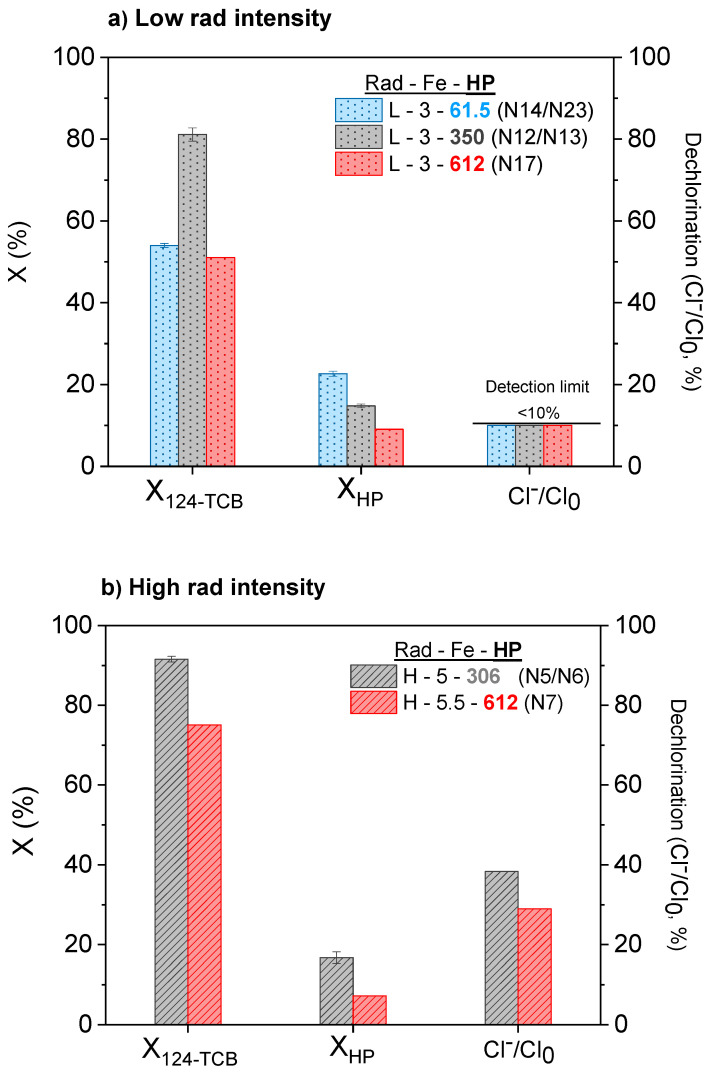
Effect of the oxidant concentration on 124-TCB and HP conversions and dechlorination degree achieved after 180 min of reaction. Experimental conditions: 124-TCB = 28 mg L^−1^, (**a**) Low rad intensity, and (**b**) High rad intensity.

The effect of the oxidant concentration (HP = 61.5–612 mg L^−1^) on the pollutant conversion and the dechlorination degree achieved was studied at three HP concentrations at low and high radiation. The results are presented in Figure 3a (Low rad intensity) and 3b (High rad intensity). The highest oxidant dosage (HP = 612 mg L^−1^) reduces the pollutant conversion and the dechlorination degree at 180 min. On the other hand, the lower oxidant concentration also yields a lower value of pollutant conversion at this time. Therefore, an optimal oxidant concentration of the oxidant agent is inferred. 

The higher the oxidant concentration, the higher the radical production (Equations (3) and (4)). However, an excess of oxidant can also enhance the unproductive consumption of hydroxyl radical, as shown in Equation (6) [58]

(6) $$H_{2}O_{2}+OH\cdot \;\to HO_{2}^{-}\cdot +H_{2}O$$

As expected, the oxidant conversion was lower as the initial hydrogen peroxide concentration increased (Figure 3a,b).

### 3.4. Response Surface Models Analysis

The Response Surface Models were applied to predict 124-TCB conversion at t = 180 min, $X_{124-TCB,pred}^{180\;\min}$. The data in Table 1 were fitted to Equation (7) for each level of radiation studied (M1: Low Radiation intensity and M2: High Radiation intensity). Variables considered were the initial concentration of hydrogen peroxide (HP, mg L^−1^) and the initial concentration of Iron (III) (Fe, mg L^−1^). Multiple regression analysis was applied to calculate the parameters *a, b, c, d, e,* and *f* in Equation (7) at each irradiation level.

(7) $$X_{124-TCB,pred\;}^{180\;\min}=a+b*HP_{o}+c*Fe_{o}+d*HP_{o}*Fe_{o}+e*HP_{o}^{2}+f*Fe_{o}^{2}$$

Fischer’s test value (F-value) and probability (*p*-value) for each model (M1 and M2) obtained from the analysis of variance (ANOVA) are presented in Table 2. First, it should be mentioned that the value of $R^{2}$ for each of both models is close to one, indicating an excellent correlation between the experimental results and those predicted by the models (corresponding to the parity plot shown in Figure S2). Moreover, the F-values were much greater than one (368.95 and 183.50 for M1 and M2, respectively), and the *p*-values < 0.0005 obtained suggested that both models are statistically significant [44].

**Figure 4:**
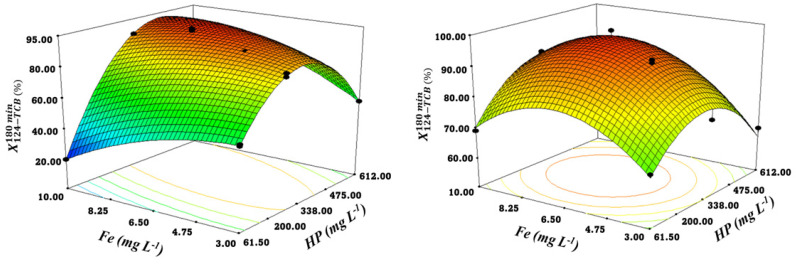
Response surface and experimental values (symbols) for 124-TCB conversion ($X_{124-TCB}^{180\;\min}$) vs HP and Fe concentrations. Experimental conditions: 124-TCB = 28 mg L^-1^, (**Left**) Low rad intensity and (**Right**) High rad intensity.

The overall effects of $HP,\;\;Fe,\;and\;HP*Fe,\;\;HP^{2},\;\;\mathrm{and}\;Fe^{2}$ M1 (Low Rad) and M2 (High Rad) response surfaces are depicted in Figure 4a and 4b, respectively. According to the shape of the surfaces, larger *Fe* and *HP* values (terms with a positive sign in the M1 and M2 models, Table 2) would result in higher 124-TCB conversion. However, the negative coefficients found for the terms $Fe_{o}^{2}\;and\;HP_{o}^{2}$ in the models are explained by the scavenging effect of hydroxyl radicals by a high dosage catalyst, Equation (5), and oxidant, Equation (6), resulting in lower 124-TCB conversions. 

The criteria for optimizing the reaction conditions consisted of maximizing the response (pollutant conversion) and maintaining the concentration of Fe in the range of study. The highest 124-TCB conversion, predicted by M1 ($X_{124-TCB,pred}^{180\;\min}=90.75\%$) and M2 ($X_{124-TCB,pred}^{180\;\min}=96.10\%$), corresponds to intermediate concentrations of the reagents (HP = 340 mg L^−1^ and Fe = 6.50 mg L^−1^ and HP = 290 mg L^−1^ and Fe = 7.10 mg L^−1^, respectively). 

Despite obtaining high pollutant conversions, the dechlorination achieved does not exceed 50% in any case (see Table 1). At this point, it is worth mentioning that the main 124-TCB oxidation byproducts detected (GC/MS) at the early stages of the reaction (traces levels) were 1,2-dichlorobenzene (12-DCB) and 1,4-dichlorobenzene (14-DCB). Volatilization can easily remove these compounds from the reaction media (they have a lower boiling point than 124-TCB [48,50]). The partial volatilization of these compounds and the presence of unidentified chlorinated byproducts could explain the low levels of dechlorination measured in some runs. In any case, higher levels of dechlorination were always achieved when High Rad conditions (Table 1) were applied. At High Rad conditions, the higher byproduct oxidation rate (12-DCB and 14-DCB) minimizes their volatilization, improving the pollutant dechlorination in the system. Therefore, the significance of radiation in the degradation of the contaminant and its dechlorination is confirmed. The oxidation of 124TCB with UV-Vis LED light was studied by Lorenzo et al. [50] using goethite as a catalyst at a neutral pH. 124-TCB conversion obtained for a similar oxidant concentration was much lower than that obtained here using the ferrioxalate complex. The toxicity of byproducts and dechlorination achieved were not studied in that work. No other studies of 124-TCB oxidation intensified by light are available to our knowledge. Using PS and alkali as the oxidants [52], oxidation times are as high as ten days to achieve 90% conversion (using higher oxidant dosages than used in this work). Therefore, it can be deduced that this compound is highly recalcitrant to oxidation, and the proposed reaction system in this work has high effectiveness.

### 3.5. Effect of Common Anions in Groundwater 

The influence of common inorganic anions found in neutral groundwater matrices ($Cl^{-}$ and $HCO_{3}^{-}$) [7] on 124-TCB conversion was evaluated at the optimal operating conditions chosen for M2 (HP = 290 mg L^−1^ and Fe = 7.10 mg L^−1^, respectively). 

**Figure 5:**
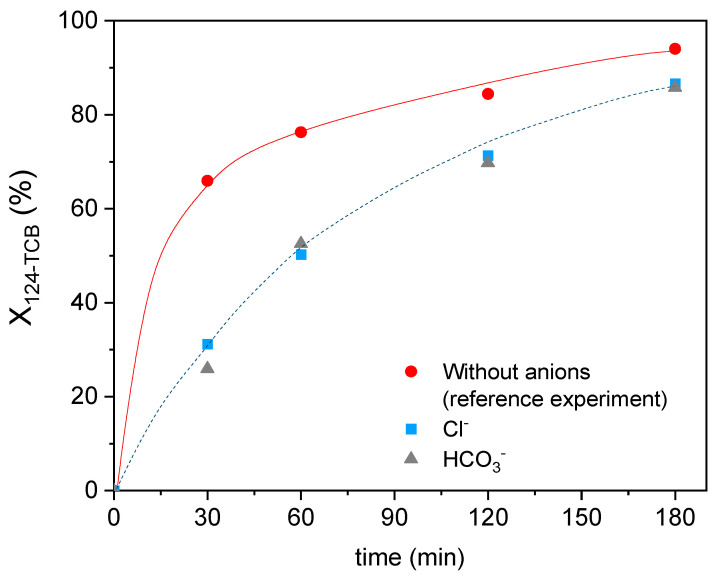
124-TCB conversion vs time for the three systems analyzed at optimum model-predicted conditions. Experimental conditions: Fe = 7.10 mg L^−1^, H_2_O_2_ = 290 mg L^−1^, 124-TCB = 28 mg L^−1^, Cl^−^ = 600 mg L^−1^, HCO_3_^−^ = 600 mg L^−1^, and High rad intensity. Experimental data as symbols. Trends as lines.

The 124-TCB conversion profile with time in the absence and presence of the studied anions is shown in Figure 5. It can be observed that the experimental conversion of the contaminant after 180 min of reaction without the anions ($X_{124-TCB,exp}^{180\;\min}=94.51\%$) was very close to the one predicted by the model ($X_{124-TCB,pred}^{180\;\min}=96.10\%$). The presence of chloride or bicarbonate decreases the pollutant conversion, although high 124-TCB conversion is still obtained at 180 min ($X_{124-TCB,exp}^{180\;\min}=85.70\%\;$). These results can be explained by the hydroxyl radical scavenging by these anions and the formation of less reactive inorganic radicals (Equation (8) and (9) [59,60].

(8) $$HCO_{3}^{-}+OH^{•}\;\overset{\;}{\to }HCO_{3}^{•}+OH^{-}\;$$

(9) $$Cl^{-}+OH^{•}\;\overset{\;}{\to }ClOH_{\;}^{•-}\;$$

In addition, the molar consumption of HP during the treatment was calculated using the Specific Consumption of the Oxidizing Agent ($\;\gamma_{HP/124-TCB}^{t}$), defined by Equation (10) [61]:

(10) $$\gamma_{HP/124-TCB}^{t}=\frac{C_{HP}^{0}-C_{HP}^{t}}{C_{124-TCB}^{0}-C_{124-TCB}^{t}}\;$$

$C_{HP}^{t}$ and $C_{124-TCB}^{t}$ are the molar concentrations of the oxidizing agent and 124-TCB remaining in the system, respectively, at a given reaction time (t); $C_{HP}^{0}$ and $C_{124-TCB}^{0}$ are the initial molar concentrations of these species. 

**Figure 6:**
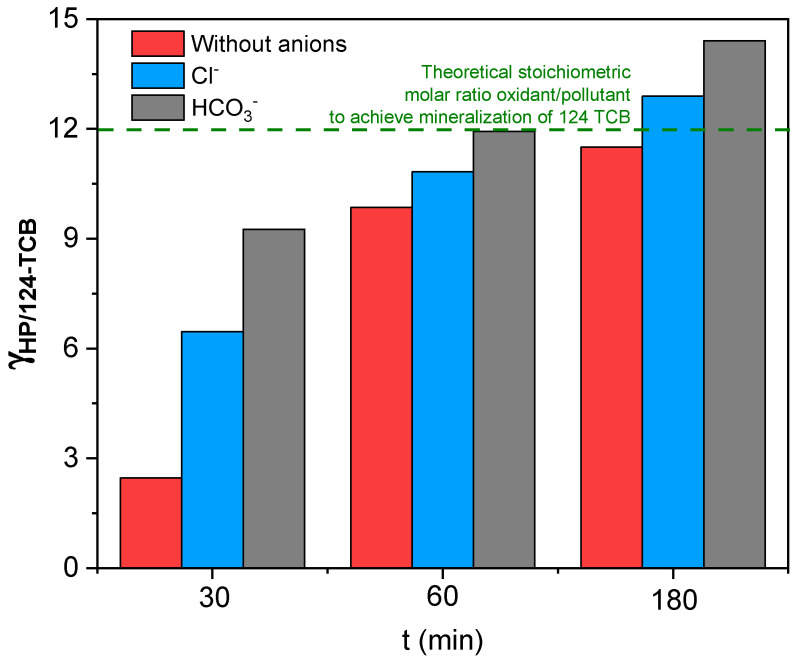
Specific consumption of the oxidizing agent with time in the absence and presence of anions. Experimental conditions: Fe = 7.10 mg L^−1^, H_2_O_2_ = 290 mg L^−1^, 124-TCB = 28 mg L^−1^, Cl^−^ = 600 mg L^−1^, HCO_3_^−^ = 600 mg L^−1^, and High rad intensity. (- - -) corresponds to the theoretical stoichiometric molar ratio oxidant/pollutant to achieve mineralization of 124-TCB (Equation (1)).

Figure 6 summarizes the results obtained from the time profile of the specific consumption of the oxidizing agent in the presence and absence of bicarbonate or chloride. As can be seen, a higher consumption of oxidizing agent per mole of contaminant removed was observed in the presence of the anions at the reaction times evaluated (30, 60, and 180 min). This unproductive consumption of the hydroxyl radicals produces not only a decrease in 124-TCB conversion but also a higher consumption of the oxidant.

For short reaction times (30 and 60 min), the consumption of oxidizing agents associated with the decomposition of 124-TCB is below the stoichiometric value for mineralization of 124-TCB (12, as shown in Equation (1)), meaning that intermediate organic compounds are generated. At 180 min of reaction, this value ($\gamma_{HP/124-TCB}^{t=180\;\min}=11.55\;mol\;HP\;mol^{-1}\;124-TCB$) was close to the stoichiometric one, probably associated with the oxidation of these intermediate compounds. The maximum oxidant consumption ($\gamma_{HP/124-TCB}^{t=180\;\min}=14.45\;mol\;HP\;mol^{-1}\;124-TCB$) was found for the $HCO_{3}^{-}$ system (t = 180 min). This value implies an additional consumption of 2.90 mol of HP per mol of contaminant removed, compared to the $\gamma_{HP/124-TCB}^{t=180\;\min}$ obtained in the absence of anions. This additional consumption is associated with the inhibitory effect of inorganic species in real groundwater. Values of $\gamma_{HP/124-TCB}^{t}$ for runs in Table 1 are also included in Table 1.

### 3.6. Toxicity Evaluation

**Figure 7:**
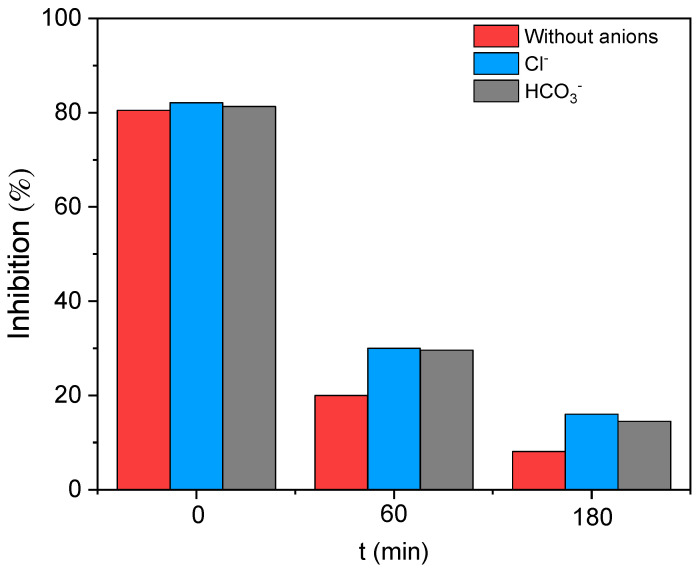
Percentage of inhibition of light emission (I (%)) after 15 min of incubation. Initial and final (t = 60 and 180 min) samples from reactions without and with anions are analyzed. Experimental conditions: Fe = 7.10 mg L^−1^, H_2_O_2_ = 290 mg L^−1^, 124-TCB = 28 mg L^−1^, Cl^−^ = 600 mg L^−1^, HCO_3_^−^ = 600 mg L^−1^, and High rad intensity.

The bioassay Microtox^®^ was applied to study the toxicity of the initial samples and samples after 60 and 180 min of reaction time, without and with bicarbonate or chloride (HP = 290 mg L^−1^, Fe = 7.10 mg L^−1^, and High Rad). The inhibition of light emission, I(%) at 15 min, samples were measured, and the results obtained are depicted in Figure 7. No inhibition of the light emitted was noticed in the solvent control toxicity measurements.

As shown in Figure 7, the maximum inhibition of light was observed at zero time (original sample) for the reference experiment (without anions, (I(%)=80.50)) and in the presence of bicarbonate (I(%)=81.35) and chloride (I(%)=82.15). Similar inhibition values are obtained at zero time, confirming that the sole presence of the anions (in the range of working concentrations) does not imply an additional toxicity to the system. After 60 and 180 min of treatment, the inhibition of light emission significantly decreased.

Therefore, it can be inferred that the toxicity is mainly attributed to the presence of 124-TCB (28 mg L^−1^). The experimentally estimated EC_50_ (concentration of the contaminant that causes a 50 % reduction of the bacteria bioluminescence) of 124-TCB in this work was 2.54 mg L^−1^ (15 min exposure), a finding with a value very close to that reported in the literature (3–3.7 mg L^−1^ [62,63]). 

For 60 min of reaction, a significant decrease in the toxicity of the three analyzed systems was observed: 75.50 %, 64.50 %, and 63.25 % for the system without the anions (I(%)=19.72), $HCO_{3}^{-}$ (I(%)=28.88), and $Cl^{-}$ (I(%)=30.19), respectively. It should be noted that in the absence of ions, this reduction is closely related to the conversion of the pollutant observed ($X_{124-TCB,exp}^{60\;\min}=78.81\%$). Therefore, it could be concluded that no toxic intermediates were present in the system. Finally, for 180 min of reaction, the inhibition obtained was less than 10% for the system without anions (I(%)=8.10) and less than 20% when bicarbonate (I(%)=14.80) or chlorides (I(%)=16.75) are present, in agreement with the lower contaminant conversion achieved in these reactions. EC50 values of less than 5 mg L^−1^ have been reported in the literature for the main 124-TCB oxidation byproducts detected (GC/MS) at the early stages of the reaction in this work (1,2-dichlorobenzene and 1,4-dichlorobenzene). Moreover, values of less than 0.05 mg L^−1^ have been reported in the literature for contaminants, such as benzoquinones and hydroxy phenols [62]. Therefore, the presence of a toxic reaction byproduct (unidentified), even in very low concentrations, could affect the inhibition of these bacteria. Therefore, high levels of toxicity removal were achieved in the three analyzed systems for optimum model-predicted operating conditions (High Rad). No toxic byproducts are noticed, and this treatment studied is a promising option for reducing chlorinated organic compounds from polluted waters. 

## 4. Conclusions

The photo–Fenton degradation of a highly toxic and persistent pollutant, 124-TCB, at a neutral pH (ferrioxalate complex as iron source) using a visible light-emitting diode lamp (Vis LED) has been proven to be an efficient treatment to oxidize the chlorinated organic pollutant. Vis LED can photo-activate the ferrioxalate complex reducing the Fe(III) to Fe(II) that initiates the radical generation by reaction with hydrogen peroxide. Moreover, high dechlorination was found, and the toxicity of the treated aqueous samples decreased remarkably. The pH was kept in the neutral region the whole time (180 min) due to the presence of the ferrioxalate complex.

Two irradiation levels were studied, finding that this variable increases the pollutant conversion, but mainly the dechlorination achieved. Variables studied at each irradiation level were hydrogen peroxide concentration (HP) and Fe(III) concentration (Fe). An increase in the concentration of either of them yielded a positive effect on pollutant abatement and dechlorination degree, explained by the higher hydroxyl radical production rate. However, this positive effect is lost at higher oxidant or catalyst dosages due to scavenging reactions among the radical, HP, and Fe(II). Therefore, an optimal oxidant and catalyst concentration value must be chosen to optimize the pollutant conversion with lower oxidant unproductive consumption. Stoichiometric, kinetic, and economic aspects should be considered to select the best conditions for each specific application. A more detailed study is required to optimize the operation conditions for each case. 

A D-optimal experimental design combined with response surface methodology (RSM) was used to evaluate and optimize the effect of the three main reaction variables simultaneously: hydrogen peroxide concentration (HP), iron dosage (Fe), and radiation intensity level (Rad). For saturated aqueous solutions of 124-TCB (28 mg L^−1^), the optimal conditions at a high radiation were 7 mg L^−1^ Fe and 310 mg L^−1^ HP. Moreover, the iron concentration selected is within the limits for water to discharge. 

The effect of two common anions ($Cl^{-}$ and $HCO_{3}^{-}$) present in neutral groundwater matrices was evaluated under optimum conditions (High Rad, HP = 290 mg L^−1^, and Fe = 7.10 mg L^−1^), obtaining a slight decrease in the 124-TCB conversion (from 94.51% to about 85% at 180 min). The inhibition of the light emitted by the *Vibrio fischeri* bacteria (*I*(%)) was evaluated in the presence and the absence of the anions. In all the cases, a significant decrease in the remaining toxicity was noticed even at 60 min, being >85% at 180 min. This reduction was proportional to the reacted 124-TCB, indicating that there were no toxic intermediates in the system, in agreement with the high dechlorination noticed.

It should be mentioned that the Vis LED source used in this research work corresponds to the range of visible wavelengths of the radiation spectrum (emission lamp peak at 470 nm). Then, the applicability of the process using renewable, abundant, and clean energy sources, namely solar radiation (the sun’s peak wavelength is between 483 and 502 nm), will transform the process into a nonexpensive, competitive method to treat this COCs present in real contaminated waters. 

## Figures and Tables

**Table 1:** Run conditions using D-Optimal experimental design for two independent variables, HP and Fe^3+^ at Low (I = 0.12 W cm^−2^) and High (I = 0.12 W cm^−2^) Rad conditions; $X_{124-TCB}^{180\;\min}$ is the analyzed response. Molar ratio Fe^3+^:Oxalate = 1:10. Results at 180 min for 124-TCB and HP conversions ($X_{124-TCB}^{180\;\min}$ and $X_{HP}^{180\;\min}$). Dechlorination $(Cl^{-}/Cl_{0}{}^{180\;\min}$) and specific consumption of the oxidant ($\gamma_{HP/124-TCB}^{180\;\min}$) are also shown. (Molecular weights: 124TCB = 184.45 g molg^−1^, H_2_O_2_ = 34 g molg^−1^, and Fe = 55.84 g molg^−1^).

Run/Level		Experimental Conditions				Results at 180 min			
Run	Level	HP mg L^−1^	Fe^3+^ mg L^−1^	Rad (I) W cm^−2^	q_w_ × 10^7^ Ecm^−2^ s^−1^	$X_{124-TCB}^{}$ (%)	$X_{HP}^{}$ (%)	$Cl^{-}/Cl_{0}{}^{}$ (%)	$\gamma_{HP/124-TCB}^{}$ mol HP mol 124-TCB^−1^
R1	N9	61.5	3	0.18	7.89	67.62	25	15	7.41
R2	N11	61.5	10	0.18	7.89	69.63	29.63	28	8.84
R3	N5	306	5	0.18	7.89	91.1	17.79	36.5	15.57
R4	N6 ^**b**^	306	5	0.18	7.89	92.05	15.72	38.32	10.39
R5	N10	350	3	0.18	7.89	75.45	18.11	31	11.04
R6	N3	350	7	0.18	7.89	98.5	21.08	39.54	12.58
R7	N1	350	10	0.18	7.89	87	13	35.57	10.27
R8	N2 ^**a**^	350	10	0.18	7.89	88.02	12.6	36.37	11.8
R9	N8	612	3	0.18	7.89	64.5	9	21.9	17.39
R10	N7	612	5.5	0.18	7.89	75.08	7.2	28.95	11.46
R11	N4	612	10	0.18	7.89	75.23	12	38.39	26.68
R12	N14	61.5	3	0.12	4.4	52.39	22.22	<10.00 *	6.69
R13	N23	61.5	3	0.12	4.4	53.45	23.09	<10.00 *	6.34
R14	N16	61.5	10	0.12	4.4	19.53	28	<10.00 *	18.97
R15	N12	250	3	0.12	4.4	82.25	14.5	<10.00 *	9.26
R16	N13 ^**c**^	250	3	0.12	4.4	80.63	15.11	<10.00 *	8.97
R17	N19	306	5	0.12	4.4	86.32	17.65	21.72	15.88
R18	N20 ^**d**^	306	5	0.12	4.4	87.54	15.19	22.9	12.69
R19	N18	350	7.5	0.12	4.4	92.12	21	26.4	37.02
R20	N21 ^**e**^	350	7.5	0.12	4.4	91.56	19.67	23.3	34.86
R21	N22	350	10	0.12	4.4	85.48	17.59	20	18.08
R22	N17	612	3	0.12	4.4	50.24	9.08	<10.00 *	22.61
R23	N15	612	10	0.12	4.4	82.56	11.22	16	22.38

**^a,b,c,d,e^** Replicates. * Dechlorination levels less than 10% negligible (experimental error).

**Table 2:** Coefficients of the Response Surface Models M1 and M2 proposed. The coefficient of variation (R^2^), Fischer’s test value (F-value), and probability (*p*-value) obtained from ANOVA tests are also shown.

Model	a	b	c	d	e	f	$R^{2}$	F-Value	*p*-Value
$X_{124-TCB,pred\;}^{180\;min}$ (M1)	35.63	0.23	1.99	0.02	$-4.29\times 10^{-4}$	−0.59	0.99	358.95	<0.0001
$X_{124-TCB,pred\;}^{180\;min}$ (M2)	19.87	0.13	15.22	$1.38\times 10^{-3}$	$-2.05\times 10^{-4}$	−1.10	0.98	183.50	<0.0005

## Data Availability

The datasets used and/or analyzed during the current study are available from the corresponding author on reasonable request.

## Supplementary Materials

The following supporting information can be downloaded at: https://www.mdpi.com/article/10.3390/ijerph19159733/s1, Figure S1: Photograph of the photoreactor used. (1) high-power collimated LED; (2) reactor; (3) power LED controller, (4) magnetic stirrer, (5) input cooling water, and (6) output cooling water Figure S2: Predicted vs Experimental 124-TCB conversion (M1 and M2); Table S1: Total nominal (Pn), real lamp power (P), irradiance (I), and the average photons flux ($q_{w})$ over the reactor window between 400–500 nm; Text S1: Section 3.2.

## References

[1] Directive E. (2013). Directive 2013/39/EU of the European Parliament and of the Council of 12 August 2013 amending Directives 2000/60/EC and 2008/105/EC as regards priority substances in the field of water policy. Off. J. Eur. Union L.

[2] van Wijk D., Cohet E., Gard A., Caspers N., van Ginkel C., Thompson R., de Rooij C., Garny V., Lecloux A. (2006). 1,2,4-Trichlorobenzene marine risk assessment with special emphasis on the Osparcom region North Sea. Chemosphere.

[3] Djohan D., Yu Q., Connell D.W. (2005). Partition Isotherms of Chlorobenzenes in a Sediment–Water System. Water Air Soil. Pollut..

[4] Lecloux A.J. (2003). Scientific activities of Euro Chlor in monitoring and assessing neutrally and man-made organohalogens. Chemosphere.

[5] Dominguez C.M., Checa-Fernandez A., Romero A., Santos A. (2021). Degradation of HCHs by thermally activated persulfate in soil system: Effect of temperature and oxidant concentration. J. Environ. Chem. Eng..

[6] Khan S., He X., Khan J.A., Khan H.M., Boccelli D.L., Dionysiou D.D. (2017). Kinetics and mechanism of sulfate radical- and hydroxyl radical-induced degradation of highly chlorinated pesticide lindane in UV/peroxymonosulfate system. Chem. Eng. J..

[7] Santos A., Fernández J., Guadaño J., Lorenzo D., Romero A. (2018). Chlorinated organic compounds in liquid wastes (DNAPL) from lindane production dumped in landfills in Sabiñanigo (Spain). Environ. Pollut..

[8] Li J.-H., Sun X.-F., Yao Z.-T., Zhao X.Y. (2014). Remediation of 1,2,3-trichlorobenzene contaminated soil using a combined thermal desorption–molten salt oxidation reactor system. Chemosphere.

[9] Wang M.-J., Jones K.C. (1994). Behaviour and fate of chlorobenzenes (CBs) introduced into soil-plant systems by sewage sludge application: A review. Chemosphere.

[10] Li H., Wang Y., Liu F., Tong L., Li K., Yang H., Zhang L. (2018). Volatile organic compounds in stormwater from a community of Beijing, China. Environ. Pollut..

[11] Bullot L., Vieira-Sellaï L., Chaplais G., Simon-Masseron A., Daou T.J., Patarin J., Fiani E. (2017). Adsorption of 1,2-dichlorobenzene and 1,2,4-trichlorobenzene in nano- and microsized crystals of MIL-101(Cr): Static and dynamic gravimetric studies. Environ. Sci. Pollut. Res..

[12] Pei Z., Li L., Sun L., Zhang S., Shan X.-Q., Yang S., Wen B. (2013). Adsorption characteristics of 1,2,4-trichlorobenzene, 2,4,6-trichlorophenol, 2-naphthol and naphthalene on graphene and graphene oxide. Carbon.

[13] Zhao Y., He P., Zhang Y.-H., Ma S. (2011). Removal of trichlorobenzene using ‘oxygen-enriched’ highly active absorbent. Environ. Technol..

[14] Andreozzi R., Caprio V., Insola A., Marotta R. (1999). Advanced oxidation processes (AOP) for water purification and recovery. Catal. Today.

[15] Đurkić T., Jazić J.M., Isakovski M.K., Maletić S., Tubić A., Dalmacija B., Agbaba J. (2019). Ultraviolet/Hydrogen Peroxide Oxidative Degradation of 1,2,3-Trichlorobenzene: Influence of Water Matrix and Toxicity Assessment. Environ. Eng. Sci..

[16] Vagi M.C., Petsas A.S. (2020). Recent advances on the removal of priority organochlorine and organophosphorus biorecalcitrant pesticides defined by Directive 2013/39/EU from environmental matrices by using advanced oxidation processes: An overview (2007–2018). J. Environ. Chem. Eng..

[17] Barbash A.M., Hoag G.E., Nadim F. (2006). Oxidation and Removal of 1,2,4-Trichlorobenzene using Sodium Persulfate in a Sorption-Desorption Experiment. Water Air Soil Pollut..

[18] Masten S.J., Galbraith M.J., Davies S.H.R. (1997). Oxidation of 1,3,5-trichlorobenzene using advanced oxidation processes. Ozone-Sci. Eng..

[19] Ormad P., Puig A., Sarasa J., Roche P., Martin A., Ovelleiro L. (1994). Ozonation of waste-water resulting from the production of organochlorine plaguicides derived from DDT and Trichlorobenzene. Ozone-Sci. Eng..

[20] Uchida H., Katoh S., Watanabe M. (1995). Photocatalytic Decomposition of Trichlorobenzene Using TiO_2_Supported on Nickel-Poly(tetrafluoroethylene) Composite Plate. Chem. Lett..

[21] Uchida H., Katoh S., Watanabe M. (1998). Photocatalytic degradation of trichlorobenzene using immobilized TiO_2_ films containing poly(tetrafluoroethylene) and platinum metal catalyst. Electrochim. Acta.

[22] Nakamura A., Hirano K., Iji M. (2005). Decomposition of Trichlorobenzene with Different Radicals Generated by Alternating Current Electrolysis in Aqueous Solution. Chem. Lett..

[23] Lin S., Su G., Zheng M., Ji D., Jia M., Liu Y. (2012). Synthesis of flower-like Co_3_O_4_-CeO_2_ composite oxide and its application to catalytic degradation of 1,2,4-trichlorobenzene. Appl. Catal. B Environ..

[24] Lin S., Su G., Zheng M., Jia M., Qi C., Li W. (2011). The degradation of 1,2,4-trichlorobenzene using synthesized Co_3_O_4_ and the hypothesized mechanism. J. Hazard. Mater..

[25] Đurkić T., Jazić J.M., Watson M., Bašić B., Prica M., Tubić A., Maletić S., Agbaba J. (2020). Application of UV-activated persulfate and peroxymonosulfate processes for the degradation of 1,2,3-trichlorobenzene in different water matrices. Environ. Sci. Pollut. Res..

[26] Oller I., Malato S. (2021). Photo-Fenton applied to the removal of pharmaceutical and other pollutants of emerging concern. Curr. Opin. Green Sustain. Chem..

[27] Ziembowicz S., Kida M. (2022). Limitations and future directions of application of the Fenton-like process in micropollutants degradation in water and wastewater treatment: A critical review. Chemosphere.

[28] Xu M., Wu C., Zhou Y. (2020). Advancements in the Fenton Process for Wastewater Treatment. Advanced Oxidation Processes—Applications, Trends, and Prospects.

[29] Parsons S. (2005). Advanced Oxidation Processes for Water and Wastewater Treatment.

[30] Ahile U.J., Wuana R.A., Itodo A.U., Sha’Ato R., Dantas R.F. (2019). A review on the use of chelating agents as an alternative to promote photo-Fenton at neutral pH: Current trends, knowledge gap and future studies. Sci. Total Environ..

[31] Polo-López M.I., Pérez J.A.S. (2020). Perspectives of the solar photo-Fenton process against the spreading of pathogens, antibiotic-resistant bacteria and genes in the environment. Curr. Opin. Green Sustain. Chem..

[32] Abdelhaleem A., Chu W. (2020). Prediction of carbofuran degradation based on the hydroxyl radical’s generation using the FeIII impregnated N doped-TiO_2_/H_2_O_2_/visible LED photo-Fenton-like process. Chem. Eng. J..

[33] Carra I., Pérez J.A.S., Malato S., Autin O., Jefferson B., Jarvis P. (2014). Application of high intensity UVC-LED for the removal of acetamiprid with the photo-Fenton process. Chem. Eng. J..

[34] de Souza Z.S.B., Silva M.P., Fraga T.J., Motta Sobrinho M.A. (2021). A comparative study of photo-Fenton process assisted by neutral sunlight, UV-A, or visible LED light irradiation for degradation of real textile wastewater: Factorial designs, kinetics, cost assessment, and phytotoxicity studies. Environ. Sci. Pollut. Res..

[35] Ahmed Y., Lu J., Yuan Z., Bond P.L., Guo J. (2020). Efficient inactivation of antibiotic resistant bacteria and antibiotic resistance genes by photo-Fenton process under visible LED light and neutral pH. Water Res..

[36] Lagori G., Fornaini C., Rocca J.P., Merigo E. (2017). Use of photo-Fenton’s reaction by 400-nm LED light for endodontic disinfection: A preliminary in vitro study on Enterococcus faecalis. J. Photochem. Photobiol. B Biol..

[37] Martínez-Pachón D., Espinosa-Barrera P., Rincón-Ortíz J., Moncayo-Lasso A. (2018). Advanced oxidation of antihypertensives losartan and valsartan by photo-electro-Fenton at near-neutral pH using natural organic acids and a dimensional stable anode-gas diffusion electrode (DSA-GDE) system under light emission diode (LED) lighting. Environ. Sci. Pollut. Res..

[38] Pliego G., García-Muñoz P., Zazo J.A., Casas J.A., Rodriguez J. (2016). Improving the Fenton process by visible LED irradiation. Environ. Sci. Pollut. Res..

[39] Checa-Fernandez A., Santos A., Romero A., Dominguez C. (2021). Application of Chelating Agents to Enhance Fenton Process in Soil Remediation: A Review. Catalysts.

[40] Zhang Y., Zhou M. (2018). A critical review of the application of chelating agents to enable Fenton and Fenton-like reactions at high pH values. J. Hazard. Mater..

[41] Conte L.O., Schenone A.V., Alfano O.M. (2016). Photo-Fenton degradation of the herbicide 2,4-D in aqueous medium at pH conditions close to neutrality. J. Environ. Manag..

[42] Giménez B.N., Conte L.O., Alfano O.M., Schenone A.V. (2020). Paracetamol removal by photo-Fenton processes at near-neutral pH using a solar simulator: Optimization by D-optimal experimental design and toxicity evaluation. J. Photochem. Photobiol. A Chem..

[43] Malato S., Fernandez-Ibañez P., Maldonado M.I., Blanco J., Gernjak W. (2009). Decontamination and disinfection of water by solar photocatalysis: Recent overview and trends. Catal. Today.

[44] Montgomery D.C. (2017). Design and Analysis of Experiments.

[45] Boutra B., Sebti A., Trari M. (2022). Response surface methodology and artificial neural network for optimization and modeling the photodegradation of organic pollutants in water. Int. J. Environ. Sci. Technol..

[46] Lojo-López M., Andrades J., Egea-Corbacho A., Coello M., Quiroga J. (2021). Degradation of simazine by photolysis of hydrogen peroxide Fenton and photo-Fenton under darkness, sunlight and UV light. J. Water Process Eng..

[47] Schenone A., Conte L., Botta M.A., Alfano O.M. (2015). Modeling and optimization of photo-Fenton degradation of 2,4-D using ferrioxalate complex and response surface methodology (RSM). J. Environ. Manag..

[48] Lide D.R. (2005). CRC Handbook of Chemistry and Physics.

[49] Jewett J.W., Serway R. (2008). Physics for scientists and engineers with modern physics. Vectors.

[50] Lorenzo D., Santos A., Sánchez-Yepes A., Conte L., Domínguez C.M. (2021). Abatement of 1,2,4-Trichlorobencene by Wet Peroxide Oxidation Catalysed by Goethite and Enhanced by Visible LED Light at Neutral pH. Catalysts.

[51] Murov S.L., Carmichael I., Hug G.L. (1993). Handbook of Photochemistry.

[52] Santos A., Fernandez J., Rodriguez S., Dominguez C., Lominchar M., Lorenzo D., Romero A. (2017). Abatement of chlorinated compounds in groundwater contaminated by HCH wastes using ISCO with alkali activated persulfate. Sci. Total Environ..

[53] (2007). Water Quality—Determination of the Inhibitory Effect of Water Samples on the Light Emission of Vibrio Fischeri (Luminescent Bacteria Test)—part 3: Method using Freeze-Dried Bacteria.

[54] Conte L.O., Schenone A.V., Alfano O.M. (2017). Ferrioxalate-assisted solar photo-Fenton degradation of a herbicide at pH conditions close to neutrality. Environ. Sci. Pollut. Res..

[55] Grčić I., Vujevic D., Koprivanac N. (2010). The use of D-optimal design to model the effects of process parameters on mineralization and discoloration kinetics of Fenton-type oxidation. Chem. Eng. J..

[56] Dean A., Voss D., Draguljić D., Dean A., Voss D., Draguljić D. (2017). Response Surface Methodology. Design and Analysis of Experiments.

[57] Myers R.H., Montgomery D.C., Vining G.G., Borror C.M., Kowalski S.M. (2004). Response Surface Methodology: A Retrospective and Literature Survey. J. Qual. Technol..

[58] Kusic H., Peternel I., Ukic S., Koprivanac N., Bolanca T., Papic S., Bozic A.L. (2011). Modeling of iron activated persulfate oxidation treating reactive azo dye in water matrix. Chem. Eng. J..

[59] De Laat J., Truong Le G., Legube B. (2004). A comparative study of the effects of chloride, sulfate and nitrate ions on the rates of decomposition of H_2_O_2_ and organic compounds by Fe(II)/H_2_O_2_ and Fe(III)/H_2_O_2_. Chemosphere.

[60] Wang J., Wang S. (2021). Effect of inorganic anions on the performance of advanced oxidation processes for degradation of organic contaminants. Chem. Eng. J..

[61] Conte L.O., Schenone A.V., Giménez B.N., Alfano O.M. (2019). Photo-Fenton degradation of a herbicide (2,4-D) in groundwater for conditions of neutral pH and presence of inorganic anions. J. Hazard. Mater..

[62] Kaiser K.L., Palabrica V.S. (1991). Photobacterium phosphoreum Toxicity Data Index. Water Qual. Res. J..

[63] Blaschke U., Paschke A., Rensch I., Schüürmann G. (2010). Acute and Chronic Toxicity toward the Bacteria *Vibrio fischeri* of Organic Narcotics and Epoxides: Structural Alerts for Epoxide Excess Toxicity. Chem. Res. Toxicol..

