# Supplementary material for: Vis LED Photo-Fenton Degradation of 124-Trichlorobenzene at a Neutral pH Using Ferrioxalate as Catalyst
Source: Int J Environ Res Public Health. 2022 Aug 7;19(15):9733. doi: 10.3390/ijerph19159733 (PMC9367996; doi:10.3390/ijerph19159733)
Supplement: Supplementary file 1 [file ijerph-19-09733-s001.zip › ijerph-1835892-supplementary.pdf]

Vis-LED photo-Fenton Degradation of 1,2,4-Trichlorobenzene at Neutral pH using Ferrioxalate as Catalyst

Leandro O. Conte[1,2]; Carmen M. Dominguez[1], Alicia Checa-Fernandez[1], and Aurora Santos[1].

[1]*Chemical Engineering and Materials Department. Chemical Sciences Faculty. Complutense University of Madrid, 28040, Madrid, Spain.*

[2]*Instituto de Desarrollo Tecnológico para la Industria Química (INTEC), Consejo Nacional de Investigaciones Científicas y Técnicas (CONICET) and Universidad Nacional del Litoral (UNL), Santa Fe, Argentina.*

Section 3.2 Text S1

Text S1"The total irradiance (I) of the lamp over the reactor windows (A) was calculated with Equation (1). Here, a UV-Vis spectrometer coupled with an optical fiber (100 µm of diameter) and using a cosine corrector (Ocean Insight, The Netherlands) was used to measure the discretized spectral irradiance ($I_{a,\lambda}$).

$$I = \int_{400}^{500} I_{a,\lambda} \cdot d\lambda \qquad (1)$$

Where *I* is the total irradiance (*W cm^{-2}*), and $I_{a,\lambda}$ is the discretized spectral irradiance (*W cm^{-2}nm^{-1}*) over de reactor window (*A* = 11 cm^2). The amount of incident energy expressed through the average photons flux in the reactor window ($q_w, Ecm^{-2}s^{-1}$) was estimated using Equation (2).

$$q_w = \int_{400}^{500} \frac{I_{a,\lambda}}{h \cdot \frac{c}{\lambda} \cdot Na} \cdot d\lambda \qquad (2)$$

Being h (*J s)* the Plank constant, c (*nm s^{-1})* the light speed, and Na (*6.022x10^{-23} mol^{-1}*) the Avogadro constant, respectively. It is worth mentioning that one Einstein (*E*) corresponds to one mole of photons.

Here, and to deliver a measure of intensity with very low uncertainty, the system (spectrometer with a fiber and cosine corrector) was radiometrically calibrated using an HL-3P-CAL lamp (Ocean Insight, The Netherlands).

The total irradiance was selected from 0.00 to 0.18 W cm^{-2}, based on previous works with the same LED lamp [50]. Different lamp nominal power (Pn) can be achieved using a Mightex BLS-13000-1E LED controller. As can be seen in Table S1**Error! Reference source not found.**, the real lamp power ($P = I \cdot A$) is close to its nominal (Pn) value over the reactor windows. Thus, showing the high energy efficiency of the LED lamp. The average photons flux in the reactor window ($q_w, Ecm^{-2}s^{-1}$) obtained using Equation (1) and Equation (2) has also been summarized in this Table S1.

Table S1. Total nominal (Pn), real lamp power (P), irradiance (I), and the average photons flux (q_w) over the reactor window between 400-500 nm.

Irradiation Level (Rad)	Pn (W)	P (W)	I (W cm^{-2})	q_w (E cm^{-2} s^{-1})
High Rad	2.07	1.95	0.18	7.89 x 10^{-7}
Low Rad	1.04	1.01	0.12	4.40 x 10^{-7}
Dark	0.00	0.00	0.00	0.00

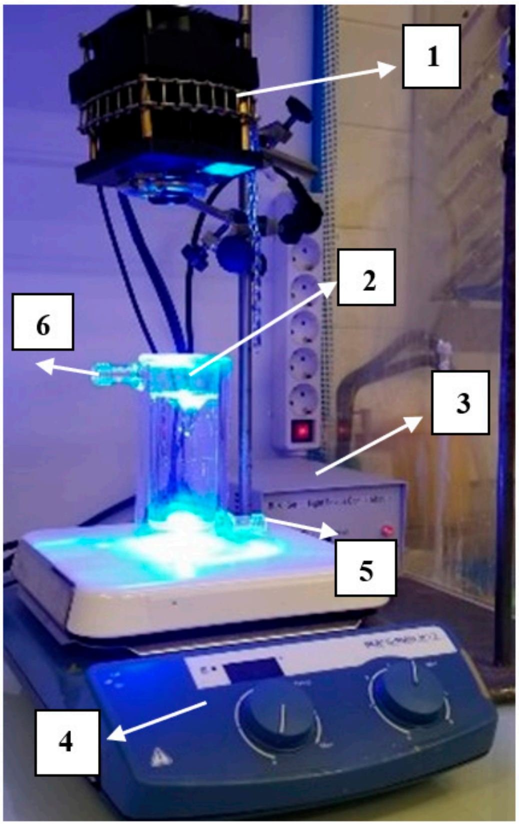

Figure S1 Photograph of the photoreactor used. (1) high-power collimated LED; (2) reactor; (3) power LED controller, (4) magnetic stirrer, (5) input cooling water, and (6) output cooling water.

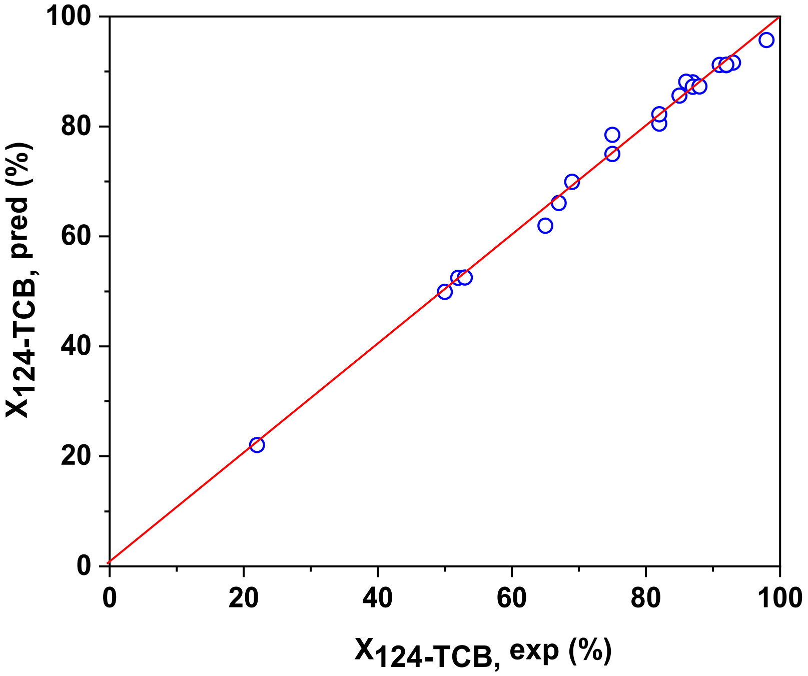

Figure S2 Predicted vs Experimental 124-TCB conversion (M1 and M2).
